# Reversible Confluent Deep White Matter Abnormalities: A New Variant of Posterior Reversible Encephalopathy Syndrome

**DOI:** 10.1155/2013/536978

**Published:** 2013-12-03

**Authors:** Yuebing Li, John Castaldo, Joshua Bemporad, Hussam A. Yacoub

**Affiliations:** ^1^Neuromuscular Center, Cleveland Clinic, Mail Code S90, 9500 Euclid Avenue, Cleveland, OH 44195, USA; ^2^Center for Advanced Health Care, Lehigh Valley Health Network, 1250 South Cedar Crest Boulevard, Suite 405, Allentown, PA 18103, USA; ^3^Medical Imaging of Lehigh Valley, Lehigh Valley Health Network, Cedar Crest & I-78, P.O. Box 689, Allentown, PA 18105, USA

## Abstract

We describe a confluent deep white matter abnormalities variant of PRES, further strengthening the notion that PRES is a disorder of radiological heterogeneity. We present 2 cases of PRES with findings of diffuse but reversible vasogenic edema located in the deep periventricular white matter regions of bilateral hemispheres without a clearly posterior distribution. We feel that this represents a rare variant of PRES on imaging, thus adding to the existing radiological spectrum for this entity. Both of our patients presented with malignant hypertension (mean arterial blood pressure of 200 mmHg) and developed neurological symptoms that included encephalopathy, seizure, headache, and vision changes. Additionally, both patients presented with significant subcortical white matter edema that improved dramatically on follow-up imaging. The clinical and radiological improvement in both patients occurred following successful blood pressure management. It is possible that the deep white matter changes of PRES are seen exclusively in the setting of severe accelerated hypertension. Our case reports reveal that, in patients with hypertensive encephalopathy, a deep white matter pattern of diffuse signal changes may not necessarily indicate chronic ischemic changes and follow-up imaging studies are essential to rule out a diagnosis of PRES.

## 1. Introduction

The clinical manifestations of posterior reversible encephalopathy syndrome (PRES) include encephalopathy, headache, visual disturbance, and seizure. In most cases, symptoms present acutely in the setting of accelerated hypertension, toxemia of pregnancy, autoimmune disorder, immunosuppressive treatment, or cancer chemotherapy. One essential feature of PRES is the presence of reversible subcortical vasogenic edema that has a predominantly posterior distribution on brain imaging [1]. Here we describe 2 cases of PRES with findings of diffuse but reversible vasogenic edema located in the deep periventricular white matter regions of bilateral hemispheres without a clearly posterior distribution. We feel that this represents a rare variant of PRES on imaging, thus adding to the existing radiological spectrum for this entity.

## 2. Case Description

### 2.1. Case No. 1

Initially found unresponsive, this 49-year-old hypertensive, hepatitis-C-positive Caucasian man presented to our emergency department with witnessed clonic tonic seizures and postictal confusion. He was treated with midazolam and fosphenytoin, followed by intubation for airway protection. Three months earlier, he presented to our hospital in a hypertensive emergency that resolved upon treatment. Home medications included aspirin, amlodipine, hydrochlorothiazide, and labetalol, but he was noncompliant.

At presentation, the patient was afebrile with an initial blood pressure of 264/168 mmHg. On evaluation he was intubated and sedated. He demonstrated minimal responses to verbal and tactile stimulation. Pupils were equal and reactive to light. Corneal and gag reflexes were intact. The serum white blood cell count (WBC) was 17,100/*μ*L, the glucose 141 mg/dL, and the creatinine 2.2 mg/dL. His electrolyte panel was normal. An electroencephalogram revealed generalized slowing without the presence of epileptiform discharges.

**Figure 1:**
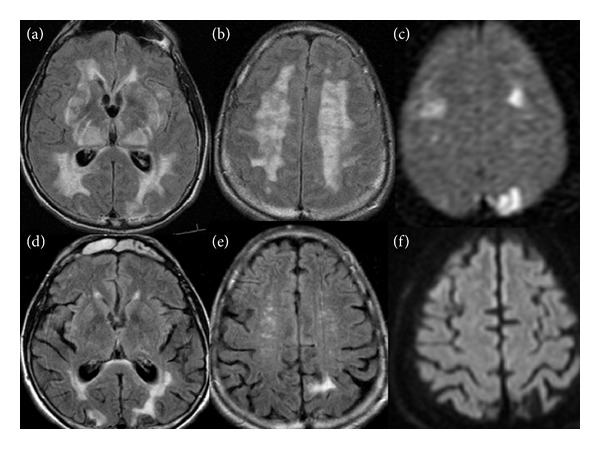
MRI findings at initial presentation (upper row) and follow-up (lower row). (a)-(b) Axial FLAIR images, taken at the levels of the hippocampi, mid lateral ventricles, and centrum semiovale, demonstrate confluent areas of abnormal high signal in the deep white matter with sparing of the subcortical white matter and overlying cortex. (d)-(e) Axial FLAIR images, at similar levels to (a)-(b), demonstrate marked decrease in the abnormal signal in the cerebral white matter. (c) shows small areas of restricted diffusion in the left greater than right medial parietal and bilateral frontal lobes, with apparent diffusion coefficient (ADC) correlation (f).

Magnetic resonance imaging (MRI) of the brain revealed confluent areas of abnormal T2 signals in the deep periventricular white matter regions extending into the subcortical areas and relatively sparing the corpus callosum. Patchy high T2 signals were also seen in the pons, cerebellum, thalamus, and basal ganglia. Small areas of restricted diffusion were detected in the left greater than right medial parietal and bilateral frontal lobes, with apparent diffusion coefficient (ADC) correlation (Figure 1). No pathological enhancement was observed with contrast administration. The imaging findings and clinical presentation were felt to be mostly consistent with PRES with an unusual distribution of white matter abnormality and vasogenic edema. Complete work-up for cardioembolic source of cerebral infarcts was unremarkable.

The patient's blood pressure was controlled with amlodipine, hydrochlorothiazide, candesartan, and labetalol; his mental status significantly improved and he was extubated the following day. Following 5 days of hospitalization, he was discharged in a stable condition. Antiepileptic therapy was discontinued at discharge. A repeat MRI after 18 months showed areas of encephalomalacia in the medial parietal and bilateral frontal lobes, which most likely represents chronic infarctions. The previously noted hyperintense T2 signals in the deep white matter, cerebellum, and pons significantly improved while small areas of hyperintense signal changes were still observed in the bilateral parieto-occipital regions (Figure 1). The patient's mental status returned to baseline and he had no further seizure activity for at least 4 years of follow-up.

### 2.2. Case No. 2

**Figure 2:**
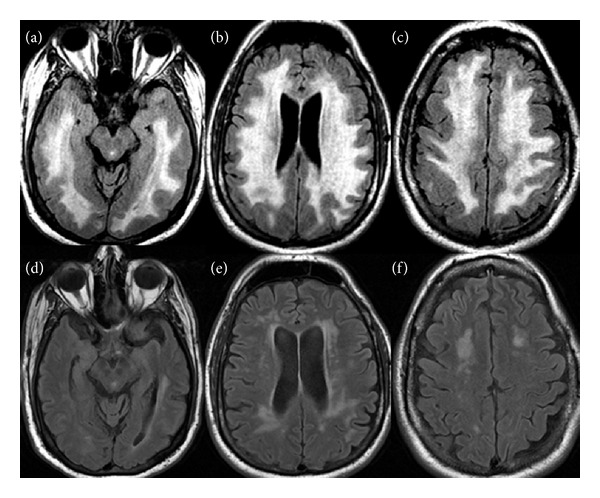
Magnetic resonance imaging findings in case 2 at initial presentation (upper row) and follow-up (lower row). (a)–(c) Axial FLAIR images, taken at the levels of the hippocampi, mid lateral ventricles, and centrum semiovale, demonstrate confluent areas of abnormal high signal in the deep white matter with sparing of the subcortical white matter and overlying cortex. (d)–(f) Axial FLAIR images, at similar levels to (a)–(c), demonstrate marked decrease in the abnormal signal in the cerebral white matter.

This 50-year-old man presented with headache, dizziness, blurred vision, shortness of breath, and leg edema. He was admitted with a diagnosis of acute renal failure. He had a long-standing history of hypertension for 20 years without treatment. He denied smoking, alcohol drinking, or use of recreational drugs. At initial presentation, he was afebrile with a blood pressure of 240/180 mmHg. He was awake and oriented to person, time, and place but had difficulties in recalling names, spelling words backwards, and following complex commands. Cranial nerve examination was normal. Motor strength examination revealed no focal weakness. The serum WBC was 13,000/*μ*L, the creatinine 2.1 mg/dL, and the glucose 148 mg/dL. An MRI of the brain demonstrated confluent T2 signal abnormalities in the bilateral deep white matter region sparing the corpus callosum (Figure 2). No significant signal abnormalities were detected in the brainstem, cerebellum, or basal ganglia. A small area of restricted diffusion was detected in the left inferior cerebellum with no corresponding signal loss on the ADC (images not shown). Contrast agent was not administered. The initial interpretation for the above imaging abnormalities was a sequel of chronic ischemic white matter disease but the clinical and radiological picture was consistent with PRES except for the distribution of white matter abnormality seen.

The patient's blood pressure was controlled with losartan, nifedipine, and metoprolol. His symptoms including cognitive impairment significantly improved following blood pressure reduction. He was discharged home after 1 week of hospitalization. Five months later, a follow-up MRI showed a marked reduction of the signal abnormalities previously seen in the deep white matter region (Figure 2).

## 3. Discussion

Posterior reversible encephalopathy syndrome is more frequently recognized with the increased availability of advanced MRI techniques. The underlying mechanism for PRES is felt to be an impairment of cerebral autoregulation and a breakdown of the blood brain barrier, likely triggered by mechanical stretching injuries from severe hypertension or chemical effects of various mediators such as cytokines from infections, toxins, or chemotherapy agents. The breakdown of blood brain barrier leads to an extravasation of fluid, protein, and other macromolecules, which may contribute to the formation of vasogenic edema. Differences in the cytoarchitecture and cerebral adrenergic innervation make the posterior circulation most susceptible to this process. Other superimposed mechanisms include vessel microdissection, arterial vasospasm, and microvascular thrombosis, consequently leading to superimposed parenchymal ischemia or hemorrhage [1].

A diagnosis of PRES is based on the following essential findings: (1) the presence of significant hypertension or other typical triggering factors such as cancer treatment or toxemia of pregnancy; (2) the occurrence of at least 1 of the following neurological symptoms: headache, seizure, visual disturbance, and mental status change; (3) the image findings of reversible predominantly subcortical vasogenic edema [2]. In the current report, both of our patients presented with malignant hypertension. Both patients developed neurological symptoms that included encephalopathy (cases 1 and 2), seizure (case 1), headache (case 2), and vision changes (case 2). Additionally, both patients presented with significant subcortical white matter edema that improved dramatically on follow-up imaging. The clinical and radiological improvement in both patients occurred following successful blood pressure management. No other etiologies, including toxic, hypoxic, infectious, or inflammatory processes, were found to explain the clinical and radiological manifestations in either patient. Therefore, based on the clinical and radiological findings as well as the treatment responses, we feel that PRES is the most fitting diagnosis for both cases. Other etiologies such as mitochondrial encephalopathy were considered in both cases, but patient characteristics, examination findings, and imaging salient features make PRES a more fitting diagnosis.

The classic MRI findings of PRES include bilateral symmetrical hyperintensities on fluid attenuated inversion recovery (FLAIR) sequence in the parieto-occipital and posterior frontal regions, with the involvement of more subcortical white matter than cortical gray matter. Such a classical pattern is typically seen in approximately 70% of patients [2]. In recent years, a variety of atypical imaging patterns of PRES have been recognized and reported, including asymmetrical, unilateral, and isolated lobar involvement pattern [3]. Other imaging findings in PRES include reversible vasogenic edema exclusively seen in the brainstem, cerebellum, or basal ganglia [4]. Spinal cord involvement has been recently reported in several PRES patients as well [5].

We describe a confluent deep white matter abnormalities variant of PRES, further strengthening the notion that PRES is a disorder of radiological heterogeneity. Other atypical imaging findings in PRES include areas of ischemia manifesting as restricted diffusion on diffusion-weighted imaging with a correlation of decreased signal on apparent diffusion coefficient, reported in as many as 24% of patients [6]. Bartynski and Boardman [7] noted the presence of areas of restricted diffusion on brain imaging in 9 of 82 (11%) patients with PRES. In our previous report [2], 14 of 59 (24%) patients with PRES demonstrated areas of restricted diffusion, consistent with a 23% incidence reported by another large series [6]. In patients with PRES, the area of ischemia is generally contained within the edematous region and can expand beyond regional arterial boundaries [8]. Based on this observation, the most feasible explanation of ischemia associated with PRES is decreased perfusion due to an elevated tissue hydrostatic pressure. Therefore, in both of our patients, the presence of small and scattered areas of ischemia within confluent areas of vasogenic edema is consistent with the radiological spectrum of PRES as previously described.

The underlying mechanism for the classical posterior dominance pattern is felt to be the paucity of sympathetic innervation in the posterior brain regions, especially in the territory of basilar artery and its branches, in comparison to the carotid and anterior cerebral territories. Normally, sympathetic activation leads to arteriolar constriction in responding to severe hypertension. A relative lack of sympathetic innervation renders the posterior regions to an increased risk of hydrostatic cerebral vasogenic edema during hypertensive crisis [9]. Our description of deep periventricular white matter pattern could be explained on the basis of the theory that the penetrating vessels supplying deep gray white matters receive scarce adrenergic innervations when compared to the superficial arteries [10].

It is worth noting that different imaging patterns of PRES were observed in specific clinical settings. For instance, brainstem involvement in PRES has been reported mainly in patients with malignant hypertension [4]. Basal ganglia involvement has been seen mainly in eclamptic patients [11]. In both cases described here, the triggering factor was malignant hypertension with mean arterial blood pressure of 200 mmHg. It is very possible that the deep white matter abnormalities of PRES are seen exclusively in the setting of severe accelerated hypertension. Our case reports reveal that, in patients with hypertensive encephalopathy, a periventricular pattern of diffuse signal changes may not necessarily indicate chronic ischemic changes and follow-up imaging studies are essential to rule out a diagnosis of PRES.
